# Delayed inflammatory mRNA and protein expression after spinal cord injury

**DOI:** 10.1186/1742-2094-8-130

**Published:** 2011-10-05

**Authors:** Kimberly R Byrnes, Patricia M Washington, Susan M Knoblach, Eric Hoffman, Alan I Faden

**Affiliations:** 1 Department of Neuroscience, Georgetown University Medical Center, Reservoir Rd, NW, Washington, DC (20057), USA; 2 Department of Anatomy, Physiology and Genetics, Uniformed Services University of the Health Sciences, Jones Bridge Road, Bethesda, MD (20814), USA; 3 Center for Genetic Medicine, Children's National Medical Center, Michigan Ave, NW, Washington, DC (20010), USA; 4 Department of Integrative Systems Biology, George Washington University School of Medicine and Health Sciences, I Street NW, Washington, DC (20037), USA; 5 Department of Anesthesiology, University of Maryland School of Medicine, S. Greene St, Baltimore, MD (21201), USA; 6 Center for Shock, Trauma and Anesthesiology Research (STAR), University of Maryland School of Medicine, S. Greene St, Baltimore, MD (21201), USA

**Keywords:** Microglia, Chronic, Inflammation, Galectin-3, Mac-2,, Microarray, NADPH oxidase, DPI

## Abstract

**Background:**

Spinal cord injury (SCI) induces secondary tissue damage that is associated with inflammation. We have previously demonstrated that inflammation-related gene expression after SCI occurs in two waves - an initial cluster that is acutely and transiently up-regulated within 24 hours, and a more delayed cluster that peaks between 72 hours and 7 days. Here we extend the microarray analysis of these gene clusters up to 6 months post-SCI.

**Methods:**

Adult male rats were subjected to mild, moderate or severe spinal cord contusion injury at T9 using a well-characterized weight-drop model. Tissue from the lesion epicenter was obtained 4 hours, 24 hours, 7 days, 28 days, 3 months or 6 months post-injury and processed for microarray analysis and protein expression.

**Results:**

Anchor gene analysis using C1qB revealed a cluster of genes that showed elevated expression through 6 months post-injury, including galectin-3, p22^PHOX^, gp91^PHOX^, CD53 and progranulin. The expression of these genes occurred primarily in microglia/macrophage cells and was confirmed at the protein level using both immunohistochemistry and western blotting. As p22^PHOX ^and gp91^PHOX ^are components of the NADPH oxidase enzyme, enzymatic activity and its role in SCI were assessed and NADPH oxidase activity was found to be significantly up-regulated through 6 months post-injury. Further, treating rats with the nonspecific, irreversible NADPH oxidase inhibitor diphenylene iodinium (DPI) reduced both lesion volume and expression of chronic gene cluster proteins one month after trauma.

**Conclusions:**

These data demonstrate that inflammation-related genes are chronically up-regulated after SCI and may contribute to further tissue loss.

## Background

Spinal cord injury (SCI) is followed by delayed secondary damage that occurs for days, weeks and even months following the initial insult [1,2]. Inflammation, including the activation and migration of microglia and macrophages, plays a significant role in this secondary injury [3-9]. Microglia are the primary immune response cells in the CNS [10] and can be activated by a number of pro-inflammatory cytokines and chemokines or other alterations in the CNS environment [11,12]. Microglia respond quickly, within minutes, to environmental changes such as increases in ATP concentration or injury [13]. After SCI, microglia are the dominant monocyte occupying the injury site through 3 days post-injury, after which macrophages begin to invade the lesion site [14]; immunocytochemically, the two cell types are indistinguishable.

We have shown that genes associated with inflammation, including those expressed primarily by microglia/macrophages, are strongly up-regulated immediately after injury and remain up-regulated for at least 7 days [15]. Further, Popovich et al. [16] has demonstrated that areas of blood-spinal cord barrier permeability 14 to 28 days post-injury are associated with OX42 (microglia/macrophage) labeling, suggesting extensive monocytic activity at delayed time points post-injury.

Our earlier work investigated the delayed up-regulation of expression of selected inflammation-related genes up to 7 days after SCI [15]; these genes included C1qB, CD53, galectin-3 and p22^PHOX^, among others. While these genes have not been studied extensively in SCI, they have all been shown to play important roles in post-injury inflammation. For example, p22^PHOX ^is a core component of the NADPH oxidase enzyme, which plays a key role in the production of reactive oxygen species (ROS). This enzyme is composed of 4 cytosolic subunits (p40^PHOX^, p47^PHOX^, p67^PHOX ^and GTP-binding protein p21-Rac1) and 2 membrane subunits (gp91^PHOX ^and p22^PHOX^) [17]. ROS and their derivatives can have severe cytotoxic effects [18,19], including induction of pro-inflammatory cytokine expression via MAPK and NFκB signaling [20]. Reduction of NADPH oxidase activity can mitigate the microglial response and reduce neuronal cell death [15,21-25]. Diphenylene iodonium (DPI), a nonspecific, irreversible inhibitor of NADPH oxidase, operates by modifying the heme component of NADPH oxidase, disrupting the ability of the enzyme to generate ROS [26,27]. DPI blocks NFκB activation in microglia, which reduces iNOS and cytokine production [24]. Inhibition of NADPH oxidase with DPI also impairs peroxynitrite production and suppresses microglial-induced oligodendrocyte precursor cell death [28].

The goal of this work was to examine the chronic expression of microglial-related genes, examining up to 6 months after SCI, and to begin to assess the relationship and function of these proteins, particularly of NADPH oxidase. The characterization of inflammatory gene expression is important for understanding the role of inflammation, including microglial and macrophage activation, in secondary injury for the development of SCI therapeutics.

## Methods

### Spinal Cord Injury

Contusion SCI was performed in adult male Sprague Dawley rats as previously described [29]. Briefly, rats (275 - 325 g) were anesthetized with sodium pentobarbital (67 mg/kg, I.P.) and mild, moderate or severe injury was induced using a weight drop method, in which a 10 g weight was dropped from 17.5, 30, or 50 mm, respectively, onto an impounder positioned on the exposed spinal cord at vertebral level T-9. Sham injured animals underwent the same experimental procedures, but received a laminectomy only, without weight drop. All experiments complied fully with the principles set forth in the "Guide for the Care and Use of Laboratory Animals" prepared by the Committee on Care and Use of Laboratory Animals of the Institute of Laboratory Resources, National Research Council (DHEW pub. No. (NIH) 85-23, 2985) and were approved by the Georgetown University IACUC.

### Expression Profiling

Animals were deeply anesthetized with sodium pentobarbital (100 mg/kg, I.P.) and decapitated 4 hours, 24 hours, 7, 14 and 28 days and 3 or 6 months after injury. A 1 cm section of the spinal cord centered at the lesion epicenter, T-9, was dissected, and immediately frozen on dry ice. Two naïve controls (rats that did not undergo any surgical procedure) were also included in the analysis.

Expression profiling was performed as described previously [15,30]. Briefly, 7 μg of total RNA was extracted using TRIzol reagent (Invitrogen, Carlsbad, CA) and used for complementary DNA (cDNA) and biotinylated complementary RNA (cRNA) synthesis. RNA was then hybridized to the Affymetrix rat U34A, B, and C arrays according to the manufacturer's protocol (Affymetrix, Santa Clara, CA). Collectively these chips include approximately 28,000 genes and ESTs. Samples were not combined; each gene chip was dedicated to a single spinal cord sample.

### Microarray Quality control

We employed stringent quality control methods as previously published [31]. Each array fulfilled the following quality control measures: cRNA fold changes between 5 to 10, scaling factor from 0.3-1.5, percentage of "present" (P) calls from 40-55%, average signal intensity levels between 900-1100, housekeeping genes and internal probe set controls showed > 80% present calls, consistent values and 5'/3' ratios were < 3.

Experimental normalization, data filtering and statistical analysis on gene expression profiles were generated with the dChip probe-set algorithm and GeneSpring software using a Welch ANOVA t-test p value <0.05 between sham and injured groups.

### Pathway Analysis

The cluster of temporally correlated genes obtained from the microarray was inputted into the GeneGo MetaCore™ pathway analysis software (St. Joseph, MI). Using the Direct Interactions, Shortest Path, and Transcription Regulation algorithms, connectivity of the gene list was obtained.

### Western Blot

At 28 days and 6 months post-injury, 4 moderate-contusion injured and 2 sham injured rats per time point were anesthetized (100 mg/kg sodium pentobarbital, I.P.) and decapitated. A 1 cm section of the spinal cord (approximately 50 mg of tissue weight) centered at the lesion epicenter, T-9, was dissected, and immediately frozen on dry ice and western blot was performed as described previously [15]. Briefly, tissue was homogenized in RIPA Buffer and centrifuged to isolate protein. Twenty-five μg of protein were run in SDS polyacrylamide gel electrophoresis and blotted onto a nitrocellulose filter. The blot was then probed with antibodies against galectin-3 (1:1000; Abcam, Cambridge, MA), progranulin (1:1000; R&D Systems, Minneapolis, MN), gp91^PHOX ^(1:1000; BD Transduction Laboratories, San Jose, CA) and p22^PHOX ^(1:1000; Santa Cruz Biotechnology, Santa Cruz, CA). Immune complexes were detected with appropriate secondary antibodies and chemiluminescence reagents (Pierce, Rockford, IL). β-actin or GAPDH were used as controls for gel loading and protein transfer. Scion Image Analysis (http://www.scioncorp.com/) was used to assess pixel density of resultant blots to compare between sham-injured and injured spinal cord tissue.

### Immunohistochemistry

At 28 days post-injury, 4 moderate-contusion injured and 2 sham injured rats per time point were anesthetized (100 mg/kg sodium pentobarbital, I.P.) and intracardially perfused with 100 ml of 0.9% saline followed by 300 ml of 10% buffered formalin. A 1 cm section of spinal cord centered at the lesion epicenter, T-9, was dissected, post-fixed in 10% buffered formalin overnight and cryoprotected in 30% sucrose for 48 hours. Standard fluorescent immunocytochemistry on serial, 20 μm thick coronal sections was performed as described previously [15]. Antibodies included NeuN (1:200; Millipore, Billerica, MA), GFAP (1:100; Promega, Madison, WI), Iba-1 (1 μg/ml; Wako, Richmond, VA), C1q (1:100; US Biologicals, Swampscott, MA), galectin-3 (6 μg/ml), p22^PHOX ^(1:200), progranulin (1:200), and gp91^PHOX ^(1:100). Appropriate secondary antibodies linked to FITC or Cy3 fluorophores (Jackson Immunoresearch, West Grove, PA) were incubated with tissue sections for 1 hour at room temperature. Slides were coverslipped using mounting media containing DAPI to counterstain for nuclei (Vector Labs, Burlingame, CA). To ensure accurate and specific staining, negative controls were used in which the primary antibody was not applied to sections from injured tissue, and only staining that labeled cells that were double-labeled with expected cell markers or had expected labeling patterns (i.e., classic microglia morphology) was confirmed as positive labeling. Immunofluorescence was detected using confocal microscopy or an AxioPlan Zeiss Microscopy system (Carl Zeiss, Inc., Thornwood, NY).

Immunofluorescence was detected and quantified in twelve 20 μm sections, selected with a random start and consistent interslice distance, using confocal microscopy as described previously [32]. In brief, the proportional area of tissue occupied by immunohistochemically stained cellular profiles within a defined target area (the lesion site and surrounding tissue) was measured using the Scion Image Analysis system using a method modified from Popovich and colleagues [33].

### NADPH Oxidase Activity Assay

At 3 and 6 months post-injury, 4 moderate-contusion injured and 2 sham injured rats per time point were anesthetized (100 mg/kg sodium pentobarbital, I.P.) and decapitated. A 1 cm section of the spinal cord (approximately 50 mg of tissue weight) centered at the lesion epicenter, T-9, was dissected, and immediately frozen on dry ice for NADPH oxidase activity assessment as previously described [34]. Briefly, tissue was homogenized in lysis buffer (50 mM Tris, 0.1 mM EDTA, 0.1 mM EGTA, 10 μg/ml aprotinin, 10 μg/ml leupeptin, 1 mM phenylmethylsulfonyl fluoride) and centrifuged at 16,000G for 15 minutes. The supernatant was then centrifuged at 100,000G for 1 hr for cell fractionation. Forty μg of protein from the membrane fraction was added to lysis buffer and NADPH oxidase buffer, along with DHE and NADPH and the plate was assessed at an emission of 485 nm and absorption of 590 nm.

### NADPH Oxidase Inhibition

For NADPH oxidase inhibition experiments, a polyethylene catheter (P-100, 1.52 outside diameter) attached to an Alzet mini-osmotic pump (Alzet, Cupertino, CA; Model 2001) was inserted into the intrathecal space at 30 minutes post-injury and advanced toward the lesion site, resting 1 - 2 mm below the lesion site, as described previously [35]. The mini-osmotic pump was loaded with either DPI (Sigma, St. Louis, MO; 100 μM in 1% DMSO in saline; n = 3) or vehicle (1% DMSO in saline; n = 3) and administered 1 μl of drug (representing 0.1 pmoles/day) or vehicle per hour for 7 days (7 day infusing pump).

### MRI Analysis

At 28 days post-injury, rats underwent magnetic resonance imaging (MRI) using a 7 Tesla 20 cm bore MRI (Bruker Biospin Billerica, MA). Rats underwent a 2D T2 weighted imaging protocol, in which the field of view was 90 × 90 mm. The TR = 3640 msec, TE = 121 msec, MTX = 256 × 256. Hyperintense areas on the 9 slices of each 90 × 90 mm MRI image was assessed using Image J analysis software, as previously described [36].

### Histological Analysis

The day after MRI imaging, spinal cord tissue was excised and processed for cresyl violet staining and lesion volume analysis as previously described {Byrnes, 2010 #3024}. Briefly, a 1 cm section of the spinal cord centered at the lesion epicenter, T9, was dissected and 20 μm thick coronal sections were collected and stained with cresyl violet. The Cavalieri method of stereology was used to estimate lesion volume, using a random start section followed by lesion volume measurement, including the cavity and surrounding damaged tissue, every 400 μm.

### Statistical Analysis

Quantitative data are presented as mean +/- standard error of the mean. Lesion volume, western blot, and immunohistochemical data were obtained by an investigator blinded to treatment group. All data were analyzed using Student's *t *test or one-way ANOVA, where appropriate. All statistical tests were performed using the GraphPad Prism Program, Version 3.02 for Windows (GraphPad Software, San Diego, CA). A *p *value < 0.05 was considered statistically significant.

## Results

### Spinal cord injury induces long term changes in expression of microglial-associated genes

**Figure 1:**
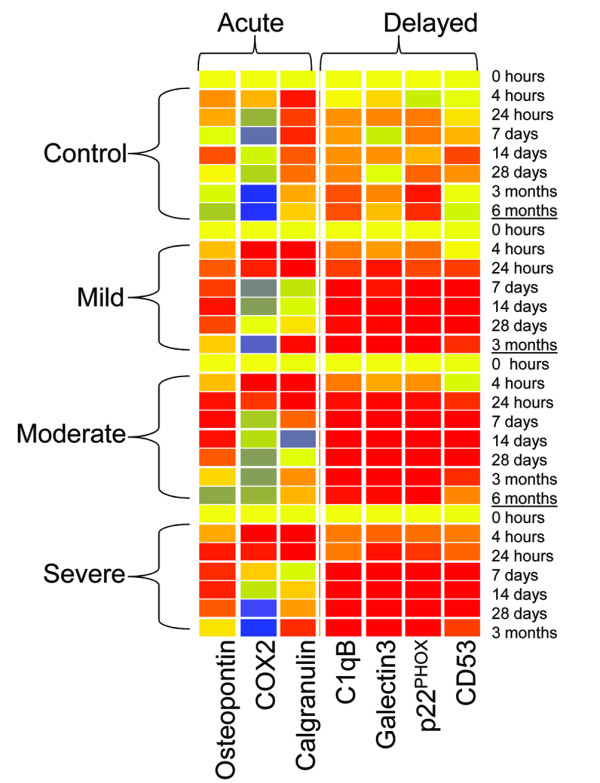
**Temporal profile patterns of the acute and delayed-expression cluster**. Time post-injury and group are represented on the y-axis; specific genes are indicated on the x-axis. Gene expression of injured and sham-injured samples are shown for the lesion epicenter after mild, moderate and severe injuries. Note the increase in expression at 4 h post-injury in the acute-expression cluster, and the more delayed increase in intensity in the delayed-expression cluster compared to that in the sham-injured group. Cool colors represent decreased expression and warmer colors indicate higher expression relative to naïve controls (yellow).

Microarray analysis was performed 4 hours, 24 hours, 7 days, 14 days, 28 days, 3 months and 6 months after mild, moderate and severe spinal cord contusion injury at T9. Gene expression data was retrieved from Affymetrix high density oligonucleotide arrays of approximately 24,000 probe sets. Our previous paper [15] detailed the expression of genes associated with microglial function up to 7 days after SCI. Analysis of chronic expression demonstrated additional expression profiles expanding upon the previous data. In this study, anchor gene temporal clustering methods identified two temporally distinct gene clusters with a correlation r^2 ^value of greater than 0.98 (Figure 1, Table 1).

**Table 1:** Abbreviated gene clusters identified after SCI

Gene Name	Description	GenBank Accession	Fold Change							
			
			4hr	24hr	72hr	7d	14d	28d	3mo	6mo
***Group 1: Acute Expression***										

Ptgs2 (COX-2)	**Anchor **Mediator of inflammatory functions, enzymatically transforms arachidonic acid into prostaglandins	S67722	2.3*	1.3*	1.7	0.9	0.6	0.9	2.9 *	2.0 *

Osteopontin	Pro-inflammatory cytokine	NM_012881	1.6	5.0*	4.7*	4.6*	0.9	0	1.3	1.7 *

S100a8 (Calgranulin)	Calcium binding protein expressed by monocytes	NM_053822	5.8*	5.5*	2.8*	0.9	0.1		1.3	0.9

***Group 2: Delayed Expression***										

C1qB	**Anchor **Component of the complement chain and binds antigen.	x71127	0.97	2.2*	3.5*	7.1*	5.9 *	7.2 *	2.2 *	1.6 *

Galectin3	Galactose specific lectin that binds IgE and extracellular matrix proteins.	JO2962	1.5*	2.4*	2.3*	10*	4.4 *	12.8*	4.0 *	4.0 *

P22/CYBa	Core component of NADPH Oxidase within phagocytes.	U18729	1.1	1.9*	2.8*	9.3*	7.8 *	4.4 *	1.7 *	1.4

OX44/CD53	Integral membrane protein that mediates signal transduction	M57276	0.9	2.2*	3.5*	6.0*	3.8 *	5.3 *	3.2 *	2.1 *

The 'acute expression' cluster, identified using the anchor genes COX-2 and Osteopontin displayed a significant increase in expression early after injury, followed by a reduction in expression by day 7 (Figure 1). There were 9 genes found to be temporally correlated within this 'acute expression' cluster (Table 2).

**Table 2:** Acute Expression Cluster

Gene Name	GenBank Accession
Ptgs2 (COX-2) **Anchor**	S67722

Osteopontin/SPP1	NM_012881

S100a8 (Calgranulin)	NM_053822

PGES	Rc_AA944447_at

EST220254	rc_AI176662_s_at

EGR1	M18416_at

RGD1564664	rc_AA945679_s_at

LOC500300	rc_AA858817_at

RGD1560812	rc_AI230381_at

Gene name and GenBank Accession number of genes correlated at 0.98 or above. EST or untranscribed sequences have not been included in this list.

**Figure 2:**
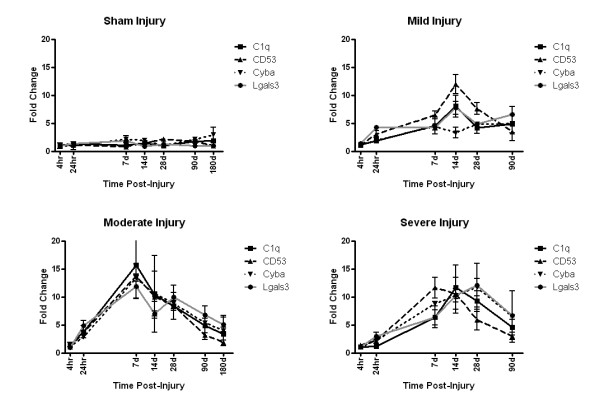
**Graphical representation of the expression of the delayed-expression cluster of genes**. A subset of genes in the delayed-expression cluster, including C1qB (C1q), CD53, p22^PHOX ^(Cyba), and galectin-3 (Lgals3) are graphed to demonstrate the fold expression change over naïve from 4 hr to 6 months post-injury. Note the similar pattern of expression within the group as well as between injury severities (Moderate, Mild, and Severe). Note also that expression of genes in the delayed-expression cluster remained up-regulated (above a 2 fold increase) through 6 months post-injury.

The 'delayed expression' cluster, identified using the C1qB anchor gene, was not up-regulated until 24 to 48 hours after injury, but then remained up-regulated throughout the study, up to 6 months post-injury (Table 1, Figure 1, 2). Expression of genes in this cluster peaked between 7 and 28 days post-injury, and remained elevated in comparison to sham-injured rats through 6 months post-injury. This expression profile was similar regardless of injury severity. Twenty-three genes were found to be temporally correlated, with an r^2 ^value of greater than 0.99 in this cluster (Table 3).

**Table 3:** Delayed Expression Cluster

Gene Name	GenBank Accession
C1qB **(Anchor)**	x71127

Fc receptor, IgG, low affinity III	M32062_g_at

Glucuronidase, beta	M13962mRNA#2_at

Similar to MS4A6B protein	rc_AI012739_at

*Galectin 3*	JO2962

Capping protein (actin filament), gelsolin-like (predicted)	rc_AA894004_at

*Granulin*	X62322_g_at

*P22/CYBa*	U18729

Tyro protein tyrosine kinase binding protein (DAP12)	rc_AI102519_at

Lysosomal-associated protein transmembrane 5	rc_AA925353_at

CD68 antigen (predicted)	rc_AI177761_at

OX44/CD53	M57276

Similar to carnosinase 1	rc_AI231438_at

Similar to MS4A6B protein	rc_AI012739_at

Lymphocyte cytosolic protein 2	rc_AI060017_at

Cytochrome b-245, beta polypeptide (^gp91PHOX^)	rc_AA894029_at

V-maf musculoaponeurotic fibrosarcoma oncogene family, protein B (avian)	rc_AI169152_at

Allograft inflammatory factor 1	U17919_s_at

Acid sphingomyelinase-like phosphodiesterase 3A	rc_AI177804_at

Gene name and GenBank Accession number of genes correlated at 0.98 or above. EST or untranscribed sequences have not been included in this list.

Genes in the 'delayed expression' cluster include genes previously identified [15], including p22^PHOX^, galectin-3, and AIF/MRF, as well as a selection of novel genes, including progranulin and gp91^PHOX^. These genes showed similar expression profiles, with expression delayed until 7 days post-injury followed by prolonged up-regulation for up to 6 months (Figure 2).

### Pathway analysis shows ample connectivity between clustered genes identified in the microarray

**Figure 3:**
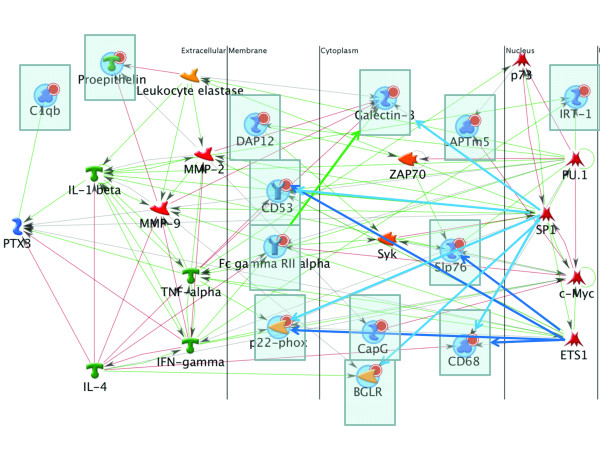
**Demonstration of pathway connections amongst genes of the delayed-expression group**. The 'Transcriptional Regulation' algorithm demonstrated a high degree of interconnection and pathway similarities amongst genes of the delayed-expression cluster (identified with blue bubbles and highlighted in boxes). Genes are organized according to compartments, with extracellularly located proteins placed on the left of the figure, and nuclear proteins on the right. Note the multiple connections between genes of the delayed-expression cluster and a few transcription factors, including ETS1 and SP1, as well as common induction pathways, such as IFNγ and TNFα. Green lines indicate positive interactions; red lines indicate negative interactions. Connections of interest, for example between transcription factors and genes of interest and direct interactions, have been highlighted.

To determine connectivity amongst the delayed expression gene cluster, GeneGo's MetaCore™ software was used to perform a pathway analysis. 'Direct Interaction' algorithm analysis revealed that several genes were directly linked. FCγRIIα was suggested to directly activate galectin-3 (indicated by a dark green arrow in Figure 3). Moving up by one interaction showed that DAP12 and Slp76 were also directly linked to FCγRIIα and galectin-3, through interaction with Zap70, Syk and SHP-1. In addition, CD53 and CD68 were linked through interactions with c-Jun.

Pathways involving the delayed expression genes were further explored using the 'Shortest Path' and 'Transcription Regulation' algorithms. These pathways suggested close interactions amongst the genes on the list, and similar transcriptional regulation (Figure 3). For example, the ETS1 transcription factor was found to be linked to CD68, p22^PHOX^, CD53, and Slip76 (indicated by dark blue arrows in Figure 3). SP1 was directly linked to CD68, BGLR, p22^PHOX^, CD53 and galectin-3 (indicated by light blue arrows in Figure 3). Similar potential induction pathways were also observed; IFNγ, for example, was associated with the expression of FCγRIIα, Slip76 and LAPTM5. Interestingly, pro-inflammatory cytokines were identified as potentially inducing the expression of several genes of the delayed expression list; for example, TNFα and IL1β may induce p22^PHOX ^and PTX3, which may induce C1qB expression. Alternatively, anti-inflammatory cytokines were associated with reduced expression of genes in the delayed expression cluster. IL4, for example, reduced FCγRIIα, CD68, and PTX3 expression. There were also instances within the identified pathways where genes on the list had functions that could induce expression of other genes on the list. For example, DAP12 and FCγRIIα were found to be associated with reduced AP1 activity, and reduction of AP1 could potentially increase galectin-3 expression.

### Galectin-3 and Progranulin protein expression confirm mRNA profiles

**Figure 4:**
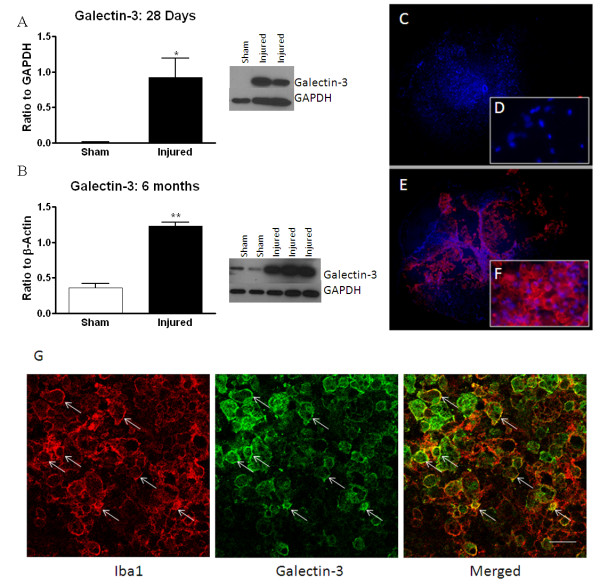
**Confirmation of galectin-3 expression at the protein level using western blotting and immunohistochemistry**. Western blotting was performed at 28 days and 6 months post-injury, and showed a significant increase in expression in moderately injured spinal cord over sham-injured tissue. Representative western blots and graphical representations are shown in A, B. Immunohistochemistry was performed at 28 days post-injury. Sham-injured tissue demonstrated no positive immunostaining for galectin-3 (C, D). Galectin-3 positive cells (labeled with a fluorescent red secondary antibody) were found throughout injured tissue at this time point, and labeled large, ameboid cells that are typical of macrophages and activated microglia (E, F). DAPI (blue) was used as a counterstain for cells. Insets show magnified areas from the white matter. Injured tissue double-labeled for microglia/macrophages (Iba-1) and galectin-3 (arrows) demonstrate correlation in expression in the merged image (G). Size bar = 500 μM (C, E); 100 μM (D, F); 75 μm (G). Bars represent mean +/- SEM.

Galectin-3 and progranulin were selected to confirm that mRNA up-regulation was paralleled by increases in protein expression using western blot and immunohistochemistry at time points from 28 days through 6 months post-injury. western blotting indicated that galectin-3 was significantly increased in injured tissue in comparison to sham-injured tissue at 28 days and 6 months post-injury (Figure 4A, B). This was confirmed with immunolabeling, which demonstrated that galectin-3 was expressed at high levels at 28 days post-injury in round/ameboid shaped cells, indicative of activated and phagocytic microglia/macrophages (Figure 4C - F). Double-label immunohistochemistry was used to confirm expression of proteins of interest in microglia/macrophages and other cell types. Galectin-3 was found to consistently label a large subset of Iba-1 positive cells, indicating microglial/macrophage expression (Figure 4F). No co-labeling was found with NeuN or GFAP, indicating that galectin-3 was not expressed in neurons or astrocytes, respectively, at 28 days after SCI (data not shown). While an assessment of the number of Iba-1/galectin-3 positive cells was not done in this study, it was clear that the majority of Iba-1 positive cells were also galectin-3 positive, and few galectin-3 positive cells were identified that were not Iba-1 positive, suggesting that galectin-3 was primarily expressed on microglia/macrophages.

**Figure 5:**
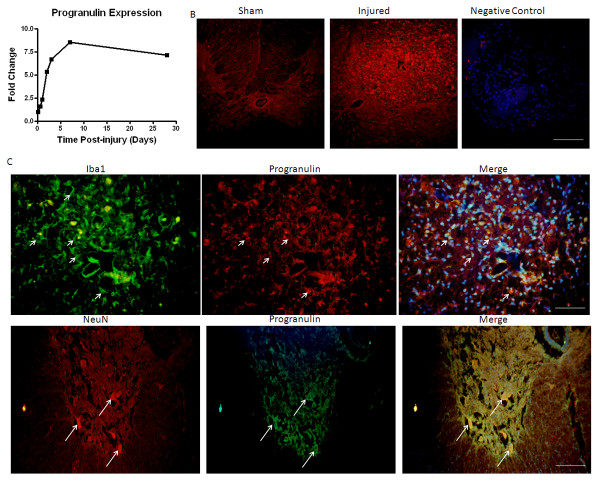
**mRNA and protein expression profile of progranulin after spinal cord injury**. Expression of progranulin was identified in the microarray and followed the expression profile of the delayed-expression cluster. Expression of progranulin peaked at 7 days post-injury and remained up-regulated through the end of the study (A). Protein expression was confirmed using immunohistochemistry at 28 days post-injury. Sham-injured tissue showed no progranulin positive cells at the lesion site (B). Injured tissue, however showed a large number of progranulin-positive cells (B). Double-labeling with Iba-1 (C) and NeuN (D) showed correlation of expression in microglia and neurons. Size bar = 200 μm (B), 100 μm (C, D).

Immunolabeling also confirmed the expression profile observed in the microarray for progranulin (Figure 5A). Progranulin was markedly increased in injured tissue at 28 days post-injury in contrast to sham-injured tissue (Figure 5B). Double-immunolabeling demonstrated that Iba-1 positive microglia/macrophages expressed progranulin (Figure 5C). However, some neurons were also positive for progranulin, as demonstrated by NeuN/progranulin positive cells (Figure 5D). Progranulin was not found in GFAP positive cells, however (data not shown).

### NADPH oxidase component expression and activity confirm mRNA expression profiles

**Figure 6:**
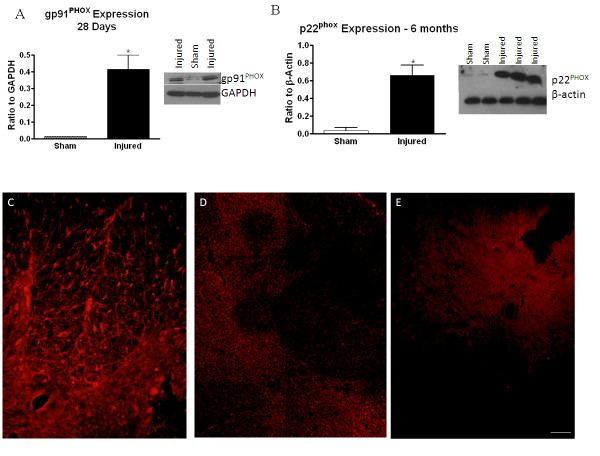
**Confirmation of components of the NADPH oxidase enzyme**. NADPH oxidase components gp91^PHOX ^(A) and p22^PHOX ^(B) were confirmed using western blotting at 28 days and 6 months post-injury. Representative western blots and graphical representation are shown. Bars represent mean +/- SEM. *p < 0.05. p22^PHOX ^expression was also confirmed with immunohistochemistry at 28 days post-injury in injured (C) and sham-injured (D) tissue. No immunolabeling was observed in negative controls where primary antibody was excluded (E), and positive labeling had typical ramified or 'bushy' appearance of microglia/macrophages. Size bar = 100 μM.

The NADPH oxidase enzyme components p22^PHOX ^and gp91^PHOX ^were both identified in the 'delayed expression' cluster. The protein products of these genes make up the membrane-bound components of NADPH oxidase enzyme, which is involved in ROS production by phagocytic cells. To confirm that the increase in gene expression is accompanied by an increase in protein expression and functional activity, western blotting, immunohistochemistry and function assays were performed for p22^PHOX ^and gp91^PHOX^. Western blot analysis of gp91^PHOX ^protein expression at 28 days post-injury indicated an increase in expression in injured tissue compared to sham tissue (Figure 6A). At 6 months post-injury, western blotting demonstrated a significant increase in p22^PHOX ^protein compared to sham, confirming the delayed expression suggested by the mRNA data (Figure 6B). Immunohistochemistry at 28 days post-injury demonstrated an increase in p22^PHOX ^immunolabeling in contrast to sham-injured tissue (Figure 6C).

**Figure 7:**
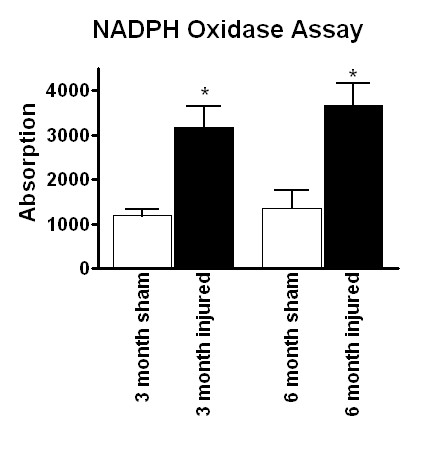
**NADPH oxidase enzyme activity profile after spinal cord injury. NADPH **oxidase enzyme activity was also assessed to confirm that gene and protein expression translated to enzymatic activity. NADPH oxidase activity was measured in sham and injured spinal cord tissue at 3 and 6 months post-injury. Bars represent mean +/- SEM. *p < 0.05.

To confirm that the protein expression was accompanied by an increase in functional activity, NADPH oxidase activity was assessed at 3 and 6 months post-injury. At both time points, NADPH oxidase activity was significantly elevated above sham levels (Figure 7), demonstrating that gene and protein expression is accompanied by functional activity.

### Inhibition of NADPH oxidase reduces lesion volume and expression of other proteins in the 'delayed expression cluster'

**Figure 8:**
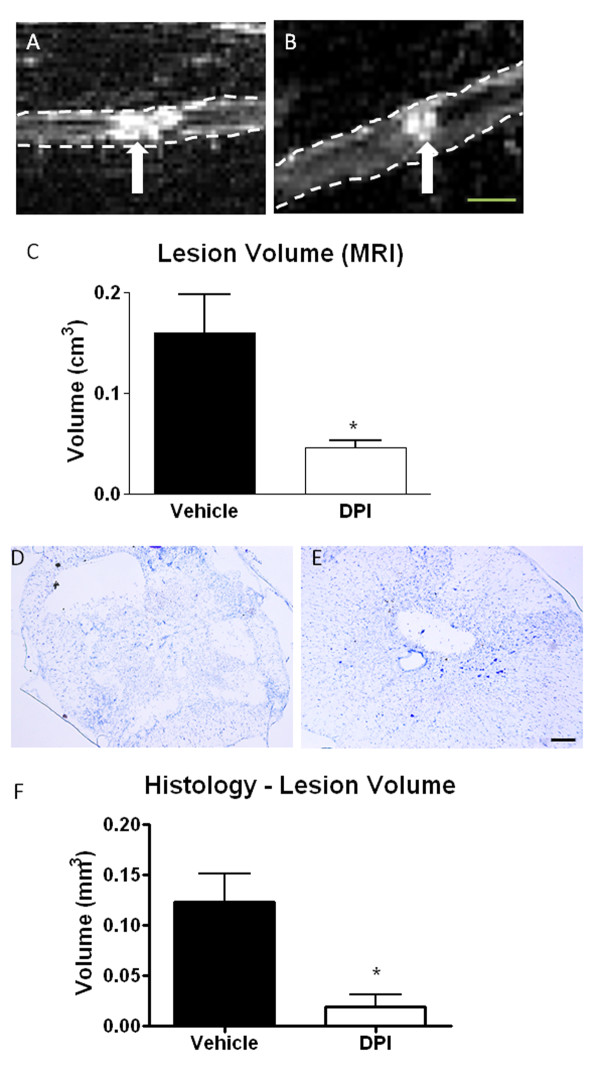
**The effect of DPI on lesion volume after spinal cord injury**. Lesion volume in vehicle and DPI-treated injured spinal cords was measured at 28 days post-injury using T2-weighted MRI and histology. Representative MRI images of the lesion (hyperintense region, arrow) are shown for vehicle (A) and DPI-treated (B) spinal cords (outlined with dotted line). Representative cresyl violet images are shown for vehicle (D) and DPI-treated (E) spinal cords as well. Quantitation of MRI (C) and histology (F) based measurements are shown. Bar size = 0.25 cm (A, B); 200 μm (D, E). Bars represent mean +/- SEM. *p < 0.05. N = 3/group.

Our data has demonstrated that NADPH oxidase expression and activity is chronically up-regulated after SCI. To determine if this enzyme plays a role in chronic expression of microglial-related inflammatory proteins after SCI, particularly those in the 'delayed expression cluster', an inhibitor of NADPH oxidase, DPI [37] was administered continuously for 7 days starting 30 minutes after injury (n = 3/group). At 28 days post-injury, all rats underwent T2-weighted MRI for assessment of post-injury lesion volume, which we have previously shown to correlate with histological measurements of lesion volume [38]. Vehicle-treated rats had a lesion volume of 0.16 +/- 0.04 cm^3 ^(Figure 8). A significant reduction in lesion volume was found in rats that received DPI treatment (Figure 8; 0.05 +/- 0.01 cm^3^). This reduction was confirmed with histology. The day after MRI, tissue was dissected and lesion volume measured in cresyl violet stained sections. Based on histological measurements, DPI-treated spinal cord had significantly (p < 0.05) smaller lesion volume than vehicle-treated spinal cord tissue (Figure 8).

**Figure 9:**
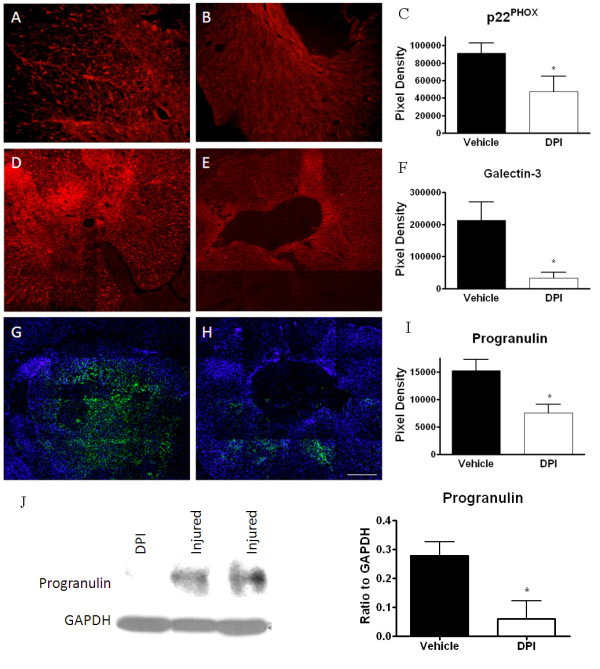
**The effect of DPI on inflammatory markers after spinal cord injury**. Inflammatory markers were compared in Vehicle (left image) and DPI-treated (right image) spinal cord injured tissue at 28 days post-injury. DPI resulted in significant reductions in p22^PHOX ^(red, A, B), progranulin (red, D, E) and galectin-3 (green, G, H) immunolabeling. DAPI-labeled nuclei are shown for contrast in G, H. Immunolabeling was quantified and graphs are shown (C, F, I). Western blotting for progranulin was performed at 28 days post-injury, and revealed a significant decrease in progranulin protein expression in the DPI treated group (J). Representative samples of the western blot are shown. Size bar = 500 μM. Bars represent mean +/- SEM; *p < 0.05.

Tissue was then assessed for multiple markers of chronic inflammation 28 days after injury. Immunohistochemistry was performed for the NADPH oxidase component p22^PHOX ^and the chronically expressed inflammatory proteins galectin-3 and progranulin. Immunolabeling for p22^PHOX^, progranulin and galectin-3 was significantly reduced by DPI treatment (Figure 9). Western blotting further confirmed the reduction of chronically expressed progranulin after DPI treatment, with a significant reduction in expression at 28 days after injury in the treated group in contrast to the vehicle group (Figure 9J).

## Discussion

This work is a continuation and expansion of our previous findings demonstrating the existence of a delayed expression cluster of inflammation-related genes [15]. Here we demonstrate that SCI results in a marked chronic up-regulation of the expression of a cluster of inflammation-related genes. Secondary injury, including chronic demyelination, also lasts for weeks to months after SCI [39]. A recent study by Naphade et al. [40] demonstrated a secondary peak of inflammation as late as 2 months post-injury. It is possible that this chronic inflammatory response may contribute to the continuation of damage in the injured cord.

Further, certain of these genes have been found to be up-regulated in other CNS injury and neurodegenerative models. For example, C1qB expression is increased in areas of demyelination in amyotrophic lateral scleroris (ALS) patients [41]. Both C1qB and galectin-3 are up-regulated after traumatic brain injury [42]. Galectin-3 has also been shown to be increased after hypoxia/ischemia from 72 hours [43] to at least 2 months post-injury [44]. Progranulin, which has recently been shown to have delayed expression after SCI [40], has also been shown to be increased in microglia in Alzheimer's [45] and ALS disease cases [46].

It is currently unclear if the protein products of the delayed expression cluster play beneficial or detrimental roles after SCI, as recent studies have sparked interest in the pro-inflammatory M1 and anti-inflammatory M2 phenotypes of microglia and macrophages [47]. While the M1/M2 status of cells expressing genes of interest was not explored in this study, it is important to note that many of the genes in the delayed expression cluster can have both advantageous and deleterious effects after injury. For example, progranulin is reportedly associated with both pro- and anti-inflammatory responses, depending upon the availability of the serine protease elastase [45,48]. Elastase is produced by cells of myeloid lineage, such as microglia; in areas with large amounts of elastase, progranulin is cleaved into granulins. Granulins, in turn, can be chemoattractants for macrophages and other inflammatory cells and can induce cytokine production [49,50]. Galectin-3 is also related to both deleterious and regenerative responses after injury. For example, it can directly induce the expression of cytokines, such as IL6 and TNFα [51]. After nerve transection, galectin-3 knockout resulted in significant increases in both the number of regrowing axons and the rate at which function was recovered [52]. Further, knockout of the gene in mice exposed to hypoxia-ischemia reduced white matter loss and markers of apoptosis [43]. However, galectin-3 knockout can reduce phagocytosis, which may impede debris clearance and regeneration [53].

The amount of interaction amongst the genes of the delayed expression cluster is also currently unknown. Interactions have been noted in the literature, including evidence of increased ROS production following C1q or galectin-3 administration to cells [54-56]. Computational pathways analysis revealed that, while there are few direct interactions between genes of the delayed-expression group, these genes may be intimately connected with similar up-stream and down-stream signaling pathways. Common transcription factors may partially explain the similar patterns of up- and down-regulation over time seen within a cluster. In this regard, a small group of transcription factors, notably ETS1 and SP1, were associated with many of the genes within the delayed-expression cluster. It is interesting to note that ETS1 and SP1 activity have not been identified previously in SCI models, despite their integral roles in the expression of several components of the inflammatory pathway [57,58], such as NAPDH oxidase components [59,60]. It is also possible that this chronic up-regulation of gene expression is a result of stimulation due to a positive feedback loop. For example, it has been shown that transcription factors, such as AP1 and SP1, are sensitive to ROS activation [61]. In fact, knockout of components of the NAPDH oxidase enzyme inhibit AP1 transcriptional activity [62].

Supporting this theory was the finding that DPI administration reduced the expression of progranulin, galectin-3 and p22^PHOX^. Moreover, T2-weighted MRI and histology revealed significantly reduced lesion volume in DPI-treated rats, suggesting that these inflammatory responses may be related to secondary injury and expansion of the lesion site after SCI. Our previous work has demonstrated that there is a significant correlation between MRI-based lesion volume and histological findings [36,38]. These data are in line with previous findings, where administration of apocynin, an alternative NADPH oxidase inhibitor, after transient middle cerebral artery occlusion reduced infarct volume [63]. Apocynin was also found to limit microglial and astrocytic activity in the hippocampus after ischemia, suggesting reductions in overall inflammatory responses [64]. It is important to note that DPI is a nonspecific inhibitor of NADPH oxidase, and has been shown to have actions on other flavin-containing enzymes. Therefore, future work will explore the effects of more specific NADPH oxidase inhibitors on SCI recovery, including motor functional recovery and axonal preservation.

In summary, these findings show that SCI in the rodent is followed by a delayed up-regulation of pro-inflammatory genes that may play a role in secondary injury. These genes are related by similar up-stream regulators, including both transcription factors and inducers. NADPH oxidase, in particular, may play a significant role in propagating chronic expression of these genes, and may serve as a target for therapeutic intervention after SCI.

## List of Abbreviations

ALS: Amyotrophic Lateral Sclerosis; cDNA: complementary deoxyribonucleic acid; cRNA: complementary ribonucleic acid; Cyba: p22^PHOX^; DPI: Diphenylene Iodinium; iNOS: inducible nitric oxide synthase; Lgals3: Galectin-3; MAPK: Mitogen activated protein kinase; MRI: Magnetic resonance imaging; mRNA: messenger ribonucleic acid; NADPH: Nicotinamide adenine dinucleotide phosphate; NFκB: Nuclear factor κ B; ROS: Reactive oxygen species; SCI: Spinal cord injury.

## Competing interests

The authors declare that they have no competing interests.

## Authors' contributions

KB carried out or directed the confirmation studies and the DPI study and drafted the manuscript. PW carried out the immunohistochemistry for both the confirmation study and the DPI study. SK carried out the microarray analysis and provided all of the microarray data. EH participated in the design of the study and completion of the microarray analysis. AIF conceived of the study, and participated in its design and coordination and helped to draft the manuscript. All authors read and approved the final manuscript.
